# Investigation of RIP140 and LCoR as independent markers for poor prognosis in cervical cancer

**DOI:** 10.18632/oncotarget.22187

**Published:** 2017-10-31

**Authors:** Aurelia Vattai, Vincent Cavailles, Sophie Sixou, Susanne Beyer, Christina Kuhn, Mina Peryanova, Helene Heidegger, Kerstin Hermelink, Doris Mayr, Sven Mahner, Christian Dannecker, Udo Jeschke, Bernd Kost

**Affiliations:** ^1^ Department of Gynaecology and Obstetrics, Ludwig-Maximilians University of Munich, 80337 Munich, Germany; ^2^ Institut de Recherche en Cancérologie de Montpellier (IRCM), INSERM U1194, Université Montpellier, F-34298 Montpellier, France; ^3^ Université Toulouse III - Paul Sabatier, F-31062 Toulouse, France; ^4^ Department of Pathology, Ludwig-Maximilians University of Munich, 81337 Munich, Germany

**Keywords:** cervical carcinoma, squamous cell carcinoma, adenocarcinoma, RIP140/NRIP1, LCoR

## Abstract

**Introduction:**

RIP140 (Receptor Interacting Protein) is involved in the regulation of oncogenic signaling pathways and in the development of breast and colon cancers. The aim of the study was to analyze the expression of RIP140 and its partner LCoR in cervical cancers, to decipher their relationship with histone protein modifications and to identify a potential link with patient survival.

**Methods:**

Immunohistochemical analyses were carried out to quantify RIP140 and LCoR expression in formalin-fixed paraffin-embedded tissue sections cervical cancer samples. Correlations of RIP140 and LCoR expression with histopathological variables were determined by correlation analyses. Survival rates of patients expressing low or high levels of RIP140 and LCoR were compared by Kaplan-Meier curves.

**Results:**

RIP140 overexpression was associated with a significantly shorter overall survival of cervical cancer patients. This effect was significant in the squamous cell carcinoma subtype but not in adenocarcinomas. RIP140 is no longer a significant negative prognosticator for cervical cancer when LCoR expression is low.

**Discussion:**

RIP140 is an independent predictor of poor survival of patients with cervical cancer. Patients with tumors expressing low levels of both RIP140 and LCoR showed a better survival compared to patients expressing high levels of RIP140. Modulation of RIP140 and LCoR may represent a novel targeting strategy for cervical cancer prevention and therapy.

## INTRODUCTION

Cervical cancer is the second most frequent female cancer and the third leading cause for cancer death in female patients worldwide [1]. The two main malignant epithelial cervical cancer types are the squamous cell carcinoma and the adenocarcinoma (about 70% and 10-25% of all cervix carcinomas, respectively) [2]. A persistent infection with high-risk human papillomavirus (HR-HPV) is the major leading cause of cervical cancer [3]. When HPV replicates, the viral E6 oncoprotein is expressed and disturbs the cell cycle [4]. The E6 oncoprotein and the E6-associated protein (E6-AP) form a complex which binds to the tumor suppressor protein p53 (an inducer of cell-cycle arrest or apoptosis [5]) and causes its proteolytic degradation [6].

The epigenetic regulation in cervical cancers can be modified through altered mechanisms such as DNA methylation and post-translational modifications of histone proteins [7]. In a recent study, we showed that histone H3 acetyl K9 (H3K9ac) and histone H3 trimethyl K4 (H3K4me3) were independent markers for poor prognosis and short overall survival (OS) in cervical cancer patients [8].

Steroid hormones act as cofactors of HPVs in the etiology of cervical cancer [9]. For instance, the regulatory region of the HR-HPV-16 contains three glucocorticoid hormone receptor response elements, which bind the glucocorticoid receptor and thereby allow viral transcription by glucocorticoids [9].

RIP140 (Receptor Interacting Protein of 140 kDa), also known as NRIP1 (Nuclear Receptor Interacting Protein 1), is a transcription coregulator of various nuclear receptors and transcription factors [10–12]. It was first identified as an ERα (estrogen receptor α) interacting protein which binds in a ligand-dependent manner to nuclear receptors and thereby limits their transactivation [13, 14]. Indeed, by means of four inhibitory domains that recruit C-terminal binding proteins and histone deacetylase, RIP140 mainly acts as a transcriptional repressor [15, 16]. More recently, an interaction of RIP140 with ERβ has also been described in ovarian cancer cells [17].

RIP140 is involved in the progression and development of cancer [18–20]. RIP140 directly interacts with E2F transcription factors, suppresses their transcriptional activity and thereby could inhibit cell proliferation [12]. However, more recently, Aziz *et al*. (2015) reported that inhibition of RIP140 expression by siRNA in breast cancer cell lines can significantly induce apoptosis and reduce cell growth [18]. In colon cancer, RIP140 has an opposing effect in comparison to breast cancer tissue as it can inhibit Wnt target gene expression and thereby decreases the ability of human colon cancer cells to proliferate [21].

Apart from RIP140, the ligand dependent corepressor (LCoR) is another transcriptional corepressor of agonist-bound nuclear receptors and other transcription factors, which also acts by recruiting histone deacetylases and C-terminal binding proteins [22–24]. Like RIP140, overexpression of LCoR represses estrogen-dependent gene expression and decreased breast cancer cell proliferation [22, 23]. Very recently, Jalaguier et al. demonstrated an interaction between RIP140 and LCoR and a strong regulation of LCoR expression by RIP140 in human breast cancer cells [22]. Interestingly, loss of RIP140 expression switches the effect of LCoR from inhibition to promotion of cell proliferation [22]. Finally, correlation of gene expression levels with clinical outcome indicated that low LCoR and RIP140 levels were associated with shorter OS in patients with breast cancer [22].

The goal of the present study was the analysis of RIP140 and LCoR expression in cervical carcinoma tissue and the correlation of their expression with patient OS. Since neither RIP140 nor LCoR has been studied in cervical cancer, this investigation represents the first analysis of these transcription factors in this pathology.

## RESULTS

### Expression of RIP140 in cervical carcinoma and correlation with histopathological variables

**Figure 1:**
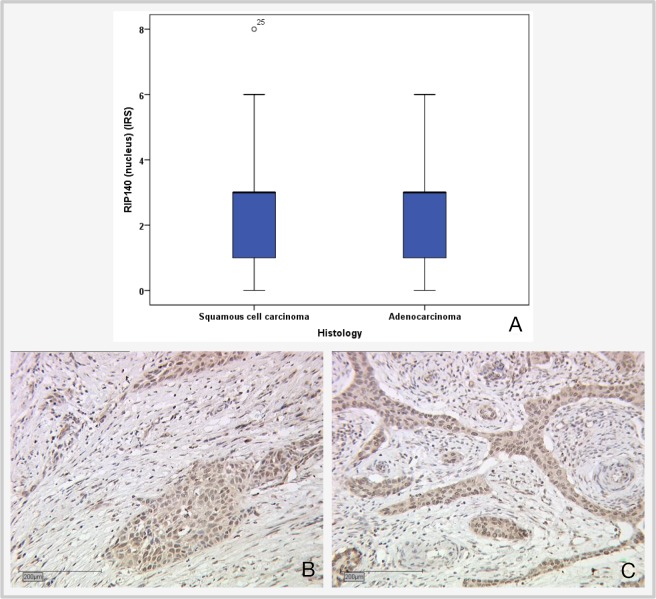
Expression of nuclear RIP140 in cervical epithelial tumor subtypes **(A)** Boxplot of RIP140 expression and histological subtype. **(B)** Squamous cell carcinoma, n=192; median RIP140 expression: IRS 3; magnification x10. **(C)** Adenocarcinoma, n=47; median RIP140 expression: IRS 3; magnification x10.

A total of 172 (71.7%) of the cervical cancer tissue samples showed positive RIP140 staining in the nucleus with a median IRS of 3 while 68 (28.3 %) did not express nuclear RIP140 (IRS=0 or 1). 10 cases could not be assessed for technical reasons. Cytoplasmic RIP140 staining was detected in 207 cases (86.3%) and 33 cases (13.7%) showed no cytoplasmic expression. Median IRS for cytoplasmic RIP140 expression was 4. The levels of nuclear RIP140 expression were assessed in the two main histological subtypes of cervical cancers. The median IRS of nuclear RIP140 expression (IRS=3) was equivalent in squamous cell carcinoma and adenocarcinoma of the cervix (Figure 1).

**Figure 2:**
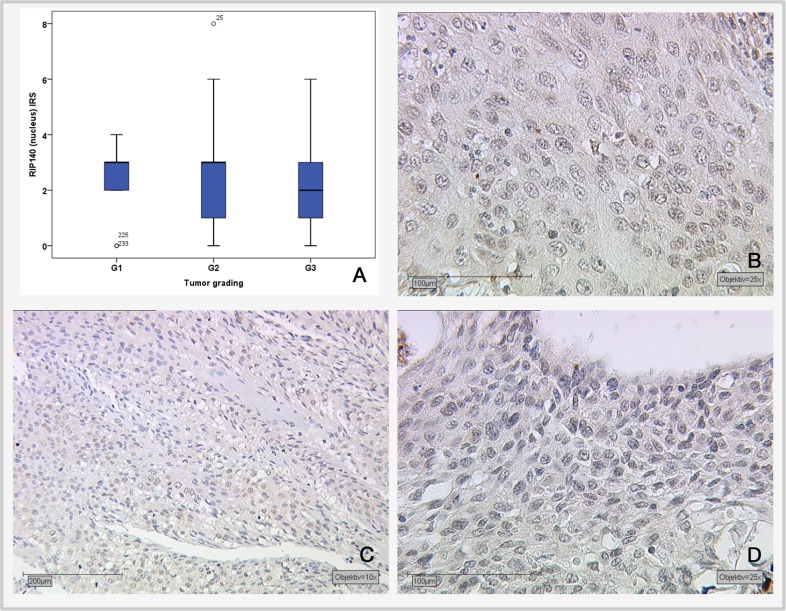
Correlation of nuclear RIP140 expression (IRS) and tumor grading **(A)** Boxplot of RIP140 expression and tumor grading. **(B)** G1-stage tumors (n=19) with median RIP140 IRS score of 3; magnification x25. **(C)** G2-stage tumors (n=137) with median RIP140 expression of 3; magnification x10. **(D)** G3-stage tumors (n=75) with median RIP140 expression of 2; magnification x25.

The Spearman test was applied for the correlation analysis of RIP140 with LCoR expression and various histopathological parameters. For positive nuclear RIP140 expression in cervical cancer tissues (with an IRS>1), a significant correlation with cytoplasmic RIP140 (p<0.001), nuclear LCoR (p=0.034), H3K9ac (p=<0.001) and tumor grading (p=0.037) were detected (Table 1). Tumor grading was negatively correlated with RIP140 expression (Spearman's rho: −0.135). Although a significant negative correlation exists between tumor grading and nuclear RIP140 expression, there is no significant difference between the different tumor grading subgroups according to the Kruskal-Wallis-test (G1 and G3: p=0.13; G1 and G2: p=0.5; G2 and G3: p=0.089) (Figure 2).

**Table 1:** Correlation analysis of RIP140 and histopathological variables

	RIP140 (nucleus) IRS>1		RIP140 (cytoplasm) IRS>1	
	Significance	Correlation coefficient	Significance	Correlation coefficient
**RIP140 (nucleus)**	-	-	<.001^***^	.552
**RIP140 (cytoplasm)**	<.001^***^	.552	-	-
**LCoR (nucleus)**	.034^*^	.137	.213	.081
**LCoR (cytoplasm)**	.370	.058	.001^**^	.213
**E6 (cytoplasm)**	.199	.083	.006^**^	.177
**P53 (cytoplasm)**	.892	.009	.256	.074
**Mutated p53 (cytoplasm)**	.588	.035	.034^*^	.137
**H3K9ac**	.001^**^	.205	.013^*^	.160
**H3K4me3**	.733	.022	.233	.077
**Tumor grading**	.037^*^	*-.135*	.060	*-.122*
**Histology**	.959	.003	.512	*-.043*
**pT**	.717	*-.024*	.321	.064
**pN**	.128	*-.098*	.723	*-.023*
**FIGO**	.986	.001	.094	.108

Significant correlations are marked with asterisks (^*^=p≤0.05, ^**^=p≤0.01, ^***^=p≤0.001). Correlation coefficients of negative correlations are marked in italics.

**Figure 3:**
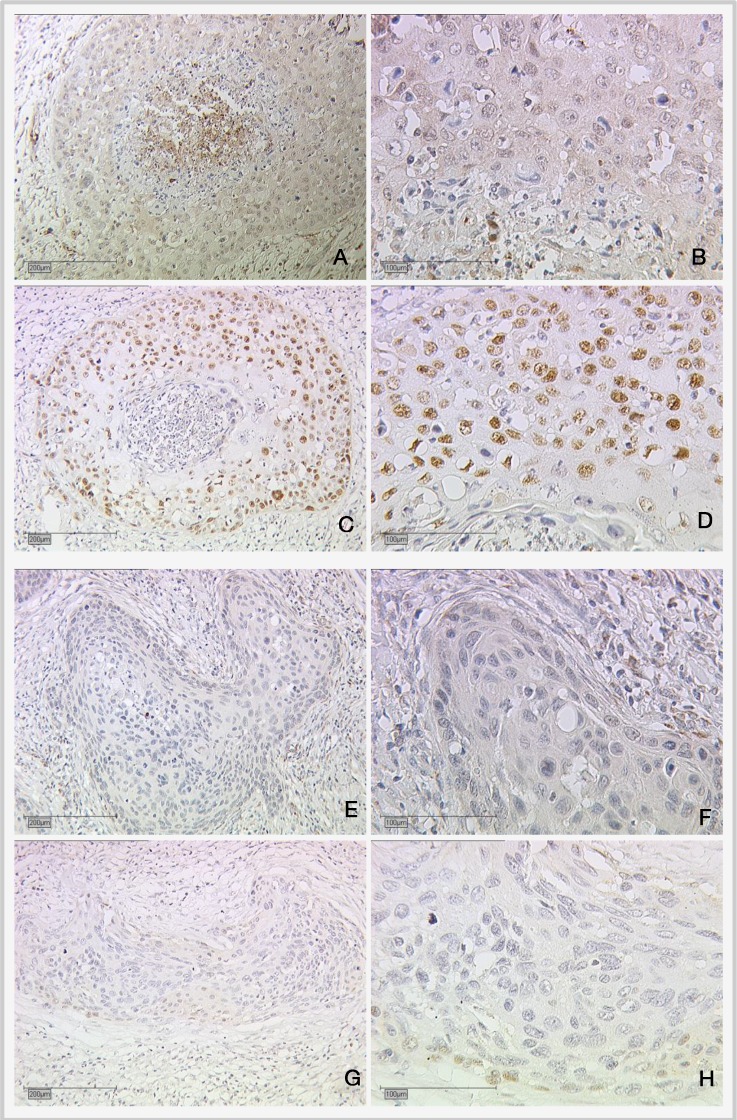
Expression of cytoplasmic RIP140 and nuclear mutated p53 in cervical cancer **(A** and **B)** High RIP140 expression (cytoplasm), SCC; **(C** and **D)** high mutated p53 (nucleus); **(E** and **F)** low RIP140 expression (cytoplasm), SCC; **(G** and **H)** low mutated p53 (nucleus). Magnifications are x10 for (A), (C), (E) and (G) and x25 for (B), (D), (F) and (H).

For cytoplasmic RIP140 expression, significant positive correlation with cytoplasmic LCoR expression (p=0.001), E6 (p=0.006) and H3K9ac (p=0.013) could be shown (Table 1). Further, a positive correlation between cytoplasmic RIP140 and mutated p53 in the nucleus was detected (p=0.034, Spearman's rho: 0.137) (Table 1). Low and high expression of mutated nuclear p53 staining and cytoplasmic RIP140 in cervical cancer are shown in Figure 3.

Correlation analysis of adjuvant radiotherapy and nuclear RIP140 expression showed no significant correlation between the two factors (p=0.894).

### LCoR staining in cervical carcinoma and correlation analysis with histopathological variables

LCoR staining in the nucleus of cervical carcinoma tissue of the collective was expressed in 47 cases (18.8%) with a median IRS of 2 and in 203 cases (81.2%) no LCoR staining could be detected. Similarly, most of the cases did not express LCoR in the cytoplasm (n=213, 85.2%) and 37 cases were positive (14.8%), with a median IRS of 4.

**Figure 4:**
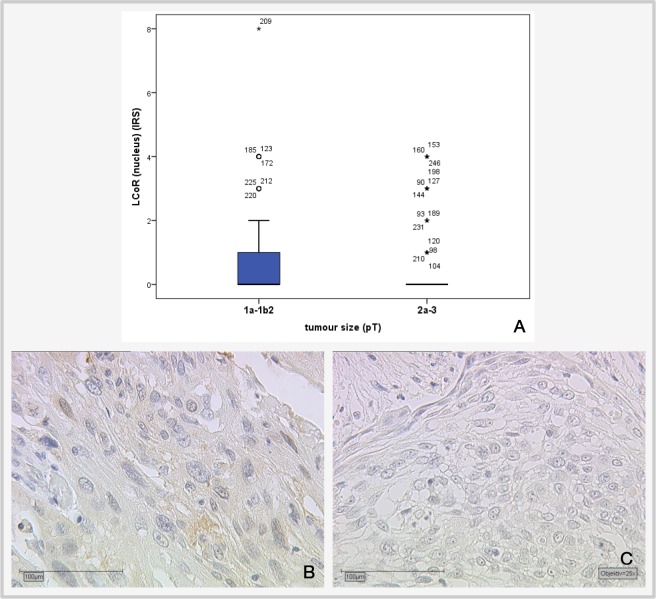
Correlation of cervical tumor size with LCoR IRS staining **(A)** Boxplot of LCoR expression and tumor size showing a significant negative correlation (p=0.039; Spearman's rho: −0.131); **(B)** median LCoR IRS in pT1a-b2 tumors = 1., magnification x25 **(C)** Median LCoR IRS in pT2a-3 cervical tumors = 0; magnification x25.

**Figure 5:**
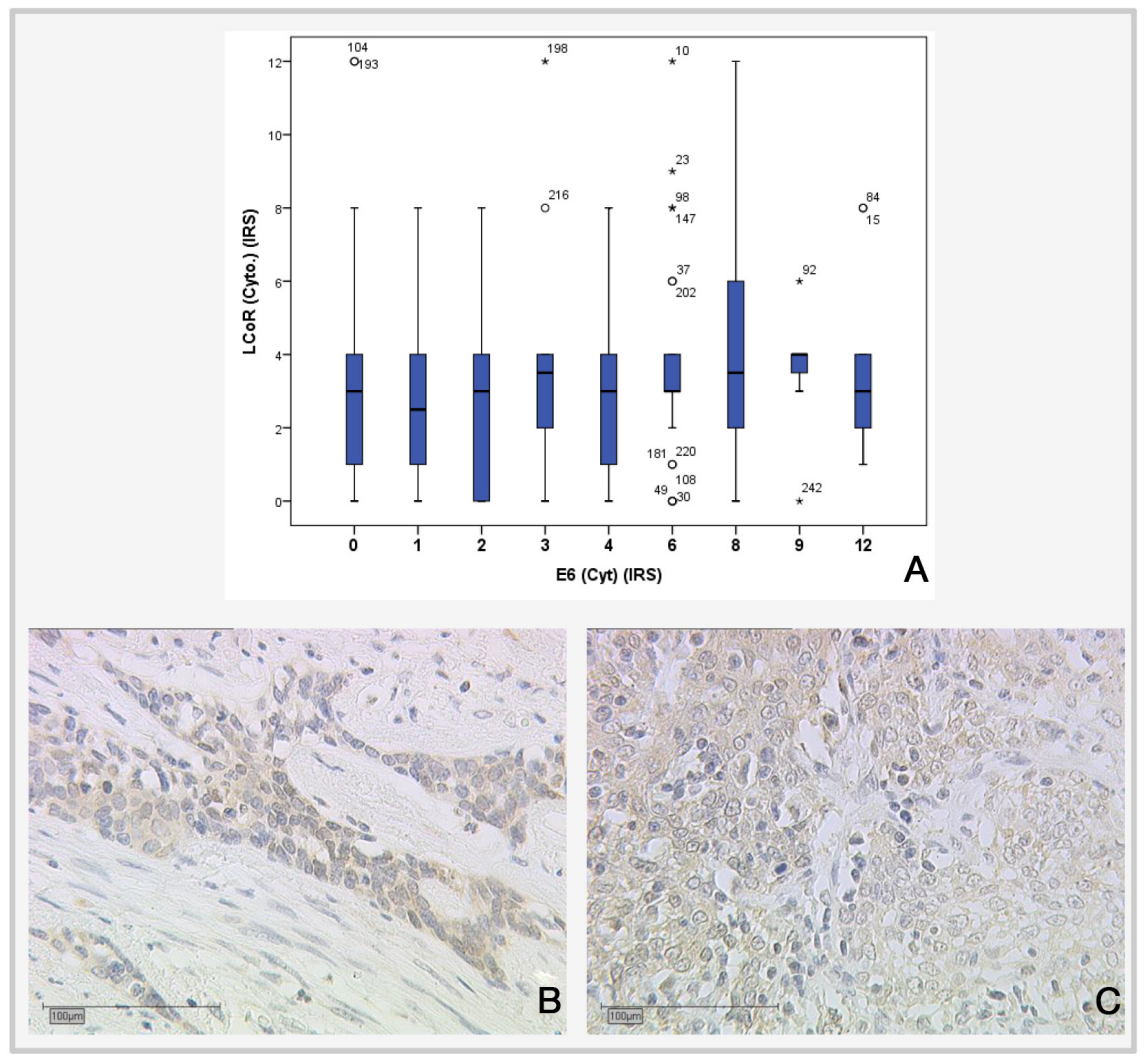
Correlation of LCoR expression with E6 expression **(A)** Boxplot showing positive correlation between LCoR and E6 expression (IRS) (p=0.022; Spearman's rho: −0.146). **(B)** LCoR expression in SCC cervical cancer, x25. **(C)** LCoR expression in SCC cervical cancer, x25.

Correlation analysis of LCoR with various histopathological parameters (Table 2) showed that nuclear LCoR expression is significantly positively correlated with cytoplasmic LCoR (p<0.001), nuclear RIP140 expression (p=0.034, Spearman's rho: 0.137) and H3K9ac (p=0.025, Spearman's rho: 0.142) and negatively correlated with tumor size (p=0.039; Spearman's rho: −0.131). A smaller tumor (pT1a-b) is associated with a higher LCoR IRS score and a larger tumor is associated with a lower IRS score (Figure 4). For cytoplasmic LCoR expression, a significant positive correlation with cytoplasmic RIP140 expression (p=0.001), E6 (p=0.022) and H3K4me3 (p=0.031) could be detected (Table 2). The correlation between cytoplasmic LCoR and E6 expression is also shown in Figure 5.

**Table 2:** Correlation analysis of LCoR expression (IRS>2) and various histopathological variables

	LCoR (nucleus)		LCoR (cytoplasm)	
	Significance	Correlation coefficient	Significance	Correlation coefficient
**LCoR (nucleus)**	**-**	**-**	<0.001^***^	
**LCoR (cytoplasm)**	<0.001^***^	.295	-	-
**RIP140 (nucleus)**	.034^*^	.137	.370	.058
**RIP140 (cytoplasm)**	.213	.081	.001^***^	.213
**E6 (cytoplasm)**	.604	.033	.022^*^	.146
**P53 (cytoplasm)**	.987	*-.001*	.560	*-.037*
**Mutated p53 (cytoplasm)**	.413	.052	.698	.025
**H3K9ac**	.025^*^	.142	.896	*-.008*
**H3K4me3**	.448	*-.048*	.031^*^	.136
**Grading**	.164	*-.088*	.456	*-.047*
**Histology**	.948	.004	.851	.012
**pT**	.039^*^	*-.131*	.623	.031
**pN**	.140	*-.094*	.329	.062
**FIGO**	.338	*-.061*	.603	.033

Significant correlations are marked with asterisks (^*^=p≤0.05, ^**^=p≤0.01, ^***^=p≤0.001). Correlation coefficients of negative correlations are marked in italics.

### Correlation of RIP140 and LCoR expression with OS and relapse-free survival of cervical cancer patients

**Figure 6:**
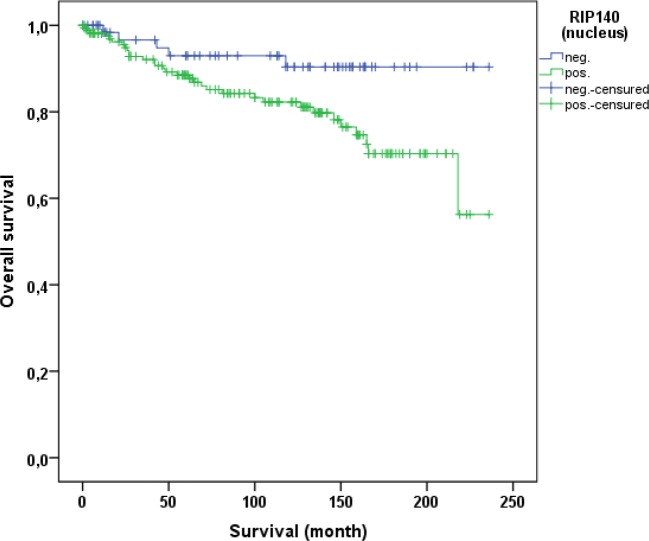
OS of patients with cervix carcinoma with a high (n=171) and a low RIP140 expression (n=68) Low RIP140 expression is associated with a longer OS in cervical cancer patients (p=0.015).

**Figure 7:**
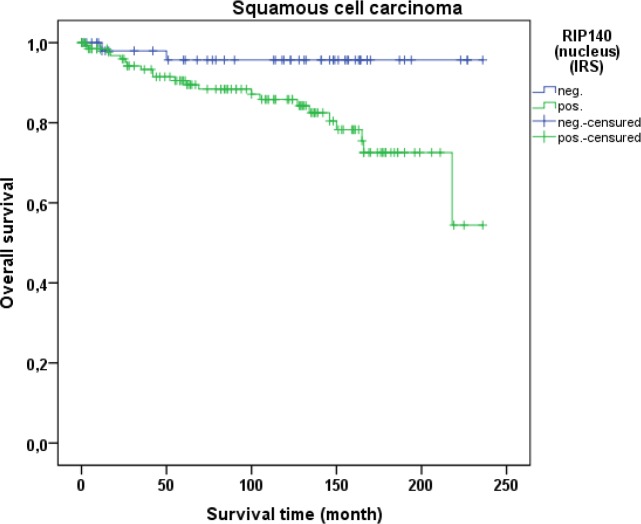
OS of patients with squamous cell carcinoma with low and high RIP140 expression There is a significant longer OS in patients with squamous cell carcinoma expressing low RIP140 levels (n=54) in comparison to tumors with high RIP140 expression (n=137) (p=0.034).

**Figure 8:**
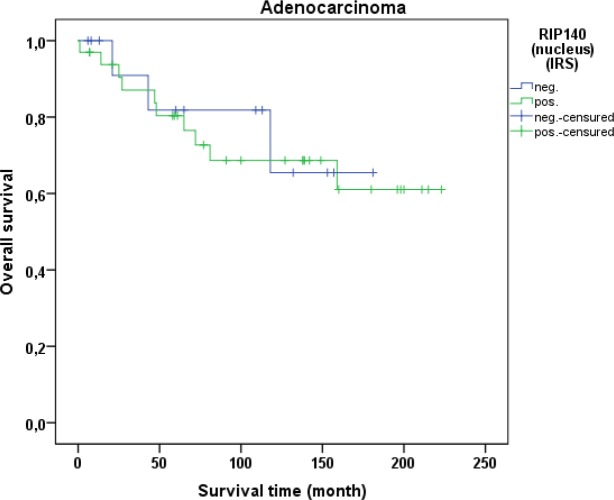
OS of patients with cervix adenocarcinomas with low and high RIP140 expression There is no significant difference in OS of patients with RIP140 negative adenocarcinoma (n=14) in comparison to RIP140 positive adenocarcinoma (n=33) (p=0.828).

Cervical cancer patients with positive nuclear RIP140 expression (n=171) were compared with patients without nuclear RIP140 expression (n=68), demonstrating that high nuclear RIP140 expression was associated with a less favorable OS in comparison to patients with low RIP140 expression (p=0.015). The significant difference is shown in the Kaplan-Meier curve in Figure 6. The OS is defined as the time period from primary surgical treatment to the time point of death in the follow up period. A receiver operating characteristic curve (ROC-curve) was used to determine the best cut-off level for high and low RIP140 expression based on the maximum difference between sensitivity and specificity. There was no significant correlation between the expression level of RIP140 in the cytoplasm (RIP140 low.: n=106; RIP140 high.: n=133) and patient OS (p=0.615). RIP140 expression, specifically in the nucleus, therefore appears to be a negative prognosticator for the OS of cervical cancer patients. The subsequent survival analysis of the two main histological subtypes showed that a significant inverse correlation of nuclear RIP140 expression with OS was observed in squamous cell carcinoma (p=0.015) (Figure 7) but not in cervix adenocarcinoma (p=0.828) (Figure 8).

**Figure 9:**
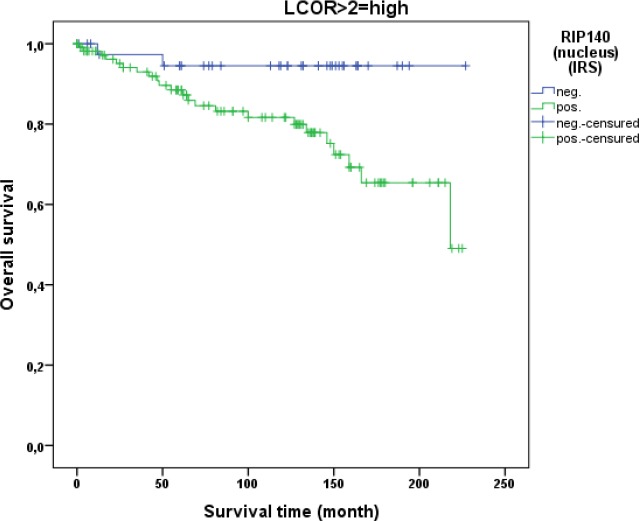
OS in patients with cervical cancer and high LCoR expression (IRS>2) classified by positive (n=113) and negative (n=40) RIP140 status RIP140 is a negative prognosticator for OS in cervical cancer patients (p=0.021) when LCoR expression is positive (IRS>2) (n=153 patients).

**Figure 10:**
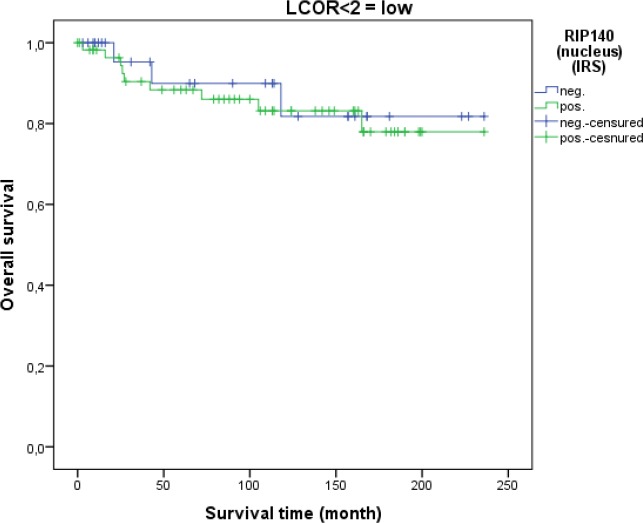
OS in patients with cervical cancer and low LCoR expression classified by positive (n=58) and negative (n=28) RIP140 status RIP140 expression is not associated with OS (p=0.710) when LCoR expression is low (IRS<2) (n=86).

LCoR expression in cervical cancer tissue was not associated with patient OS (p=0.329). This was also the case when squamous cell carcinoma and cervix adenocarcinoma were separately analyzed. As for RIP140, a ROC-curve was used to determine the cut-off level for low and high nuclear LCoR expression. Very interestingly, RIP140 is a negative prognosticator for OS of cervical cancer patients when LCoR expression is high (IRS>2) (p=0.021 - Figure 9) but not when nuclear LCoR expression is low (Figure 10). The longest OS outcome could be observed in patients with high RIP140 expression and LCoR expression being low (n=28) (Figure 10).

There is no significant difference in progression regarding LCoR/RIP140 expression. There was a trend for a shorter relapse-free survival in patients with nuclear LCoR IRS>0.5 expression in the primary cervical tumor (p=0.081) (Supplementary Figure 1).

### Multivariate cox regression analysis

Multivariate Cox regression analysis was performed to test which histopathological variables including age, FIGO-classification, histology, tumor size (pT), nodal status (pN), tumor differentiation grade, RIP140 and LCoR status were independent prognosticators for OS in our cohort of patients with cervical cancers. RIP140 expression with an IRS>1 (p=0.014), histology (p=0.002), tumor size (p=0.005) and lymph node status (p=0.020) were independent prognosticators for patient OS (Table 3). No significant effect could be seen for the other histopathological variables.

**Table 3:** Cox-regression of significant histopathological variables regarding OS in cervical cancers

	Significance	Hazard ratio Exp (B)	Lower 95% Cl of Exp(B)	Upper 95% Cl of Exp(B)
**Tumor size (pT)**	.005^**^	1.264	1.072	1.492
**Histology**	.002^**^	3.076	1.521	6.220
**RIP140 (nucleus) (IRS>1)**	.014^*^	3.385	1.285	8.918
**Nodal status (pN)**	.020^*^	2.417	1.151	5.076

Significant correlations are marked with asterisks (^*^=p≤0.05, ^**^=p≤0.01, ^***^=p≤0.001).

## DISCUSSION

Patients with breast cancer where RIP140 is expressed at high and LCoR at low levels, show a better survival rate. Our data show that, in cervical cancer biopsies, expression of RIP140 is associated with poor prognosis. In line with these observations, a previous study reported a 5.84-times enhanced expression of the *NRIP1* gene at the mRNA level in cervical cancer compared to normal tissue [25]. Moreover, integration of viral DNA into the host genome can lead to the disruption of invaded genes and can consequently play a role in the process of HPV carcinogenesis in HPV-associated cervical cancer. Recently, Olthof and colleagues (2015) identified an integration site of HPV16 within the *NRIP1* gene [26]. They showed that viral E2, E6 and E7 gene expression proved to be independent of the number of integration sites and viral load, hence integration might not affect viral gene expression [26].

RIP140 targets different pathways that are relevant for the development of cervical cancer such as estrogen receptor (ER) signaling [19]. Indeed, increased estrogen levels (through the usage of oral contraceptives or repeated parity) lead to an increased risk of cervical cancer in HPV-infected women [27]. Steroid hormones are able to increase the transcription of HPV oncogenes leading to an increased viral persistence [28] and to the degradation of p53 which might favor tumorigenesis through disruption of the normal cell cycle [29].

In addition to its influence on ER, RIP140 inhibits the transactivation potential of E2Fs transcription factors [12]. E2Fs are regulators of genes required for cell cycle progression, DNA replication, apoptosis and cell differentiation [30]. Srivastava et al. (2014) identified E2F as a potential biomarker that is assumed to regulate the transcription of a group of genes associated with cervical cancer and might thereby act as a potential molecular target for the treatment of this malignancy [31]. The inactivation of E2F repressor-Rb by HPV E7 leads to a deregulation of E2F activity and consequently to increased levels of proteins whereby transcriptional regulation is controlled [32, 33]. Genes that are involved in invasive cervical carcinoma are therefore presumably under E2F regulation [32].

Furthermore, RIP140 is involved in Wnt-signaling [34]. In colon cancer, RIP140 has a negative impact on Wnt/β-catenin target genes and thereby inhibits epithelial cell progression, cell proliferation and tumor growth [19, 21]. This contradictory effect of RIP140 in colon cancer compared to its effect on breast or cervical cancers indicates the complex roles of RIP140 on cell growth and tumor development in different tissues [18]. Depending on the tissue and the physiological or pathophysiological condition, are the corepressors RIP140 and LCoR able to function differently [24]. A participation of the Wnt/β-catenin signaling pathway in HPV-related cancers and the possible mechanisms by which the oncoproteins E6 and E7 activate this pathway has been demonstrated [35]. The Wnt/β-catenin pathway can regulate cell proliferation and apoptosis in cervical carcinomas and seems to be important for cervical oncogenesis [36].

In our correlation analysis, we showed that cytoplasmic RIP140 expression was positively correlated with cytoplasmic virus-specific oncoprotein E6 (p=0.006) and mutated p53 in the cytoplasm (p=0.034). Cytoplasmic LCoR expression also correlated significantly with E6 expression (p=0.022), but no significant correlation could be identified between LCoR and p53. The oncoproteins E6 and E7 are involved in the development of HPV-induced cervical cancer [37]. E6 protein interacts with the E3 ubiquitin-protein ligase, resulting in the proteolysis of p53 protein [38]. Mutations of p53 are genetic alterations in different human malignancies where the ability of the protein to bind to its target DNA sequence is destroyed and transcriptional activation is reduced [39, 40]. In a previous study, we could show a significant advantage of nuclear p53 protein expression (p=0.024) on the OS of cervical cancer patients [37].

In our current study, we observed a positive correlation between mutated p53 in the cytoplasm and cytoplasmic RIP140 which is associated with a worse prognosis in cervical cancer patients. As p53 can only function in the nucleus [37], we may speculate that p53 is kept in the cytoplasm and therefore correlated with the negative prognosticator for cervical cancer, RIP140, when they interact with each other. Interestingly, only the mutated form of p53 showed a negative correlation with E6 in the cytoplasm (p=0.028, Spearman's rho: −0.140) (Stiasny et al. (July 2017) in print at Oncology Letters “The role of E6 oncoprotein, p53, p16, MDM2 and Galectin-3 for the clinical outcome of cervical cancer patients”). In our case, E6 has an influence on the expression of mutated p53 in the cytoplasm and RIP140 correlates with mutated p53 in the cytoplasm. Therefore, E6 may be involved in the RIP140/mutated p53 correlation.

Finally, post-translational modifications like acetylation play major roles in controlling the repressive activity of RIP140 [41]. The transcriptional contortion in cancer induced through expression changes of co-repressors is altered by the actions of histone modifying enzymes [42]. In a recent study, we could show that the histone protein H3K4me3 was correlated with poor prognosis in cervical cancer patients and is an independent marker for relapse-free survival [8]. Moreover, the histone protein H3K9ac was found to be an independent marker of OS in cervical cancer patients [8]. In the present work, we demonstrated a significant correlation of H3K9ac levels with nuclear (p<0.001) and cytoplasmic (p=0.013) RIP140 expression as well as with nuclear LCoR expression (p=0.025). In addition, cytoplasmic LCoR levels were correlated with H3K4me3 (p=0.031). The positive correlation of RIP140 with H3K9ac levels is in line with our findings as we show that RIP140 is associated with a less favorable OS in cervical cancer patients just as the histone protein modification. It is already known that RIP140 directly interacts with HDACs [15, 16]. It has been proposed that this interaction might sequester HDACs out of their target sites and could therefore explain part of the positive effects that RIP140 exerts on gene expression such as when the transcription is driven by the transcription factor Sp1 [15]. Such a sequestration of HDACs might lead to an increase in H3K9 acetylation. Alternatively, H3K9ac marks on the genome (which are essentially related to transcriptional activation) could be linked to a global increase in gene expression including that of the *RIP140* and *LCOR* genes, thus explaining the correlations observed in the present work.

In this study, we also demonstrated that RIP140 expression (IRS>1) is associated with poor OS of patients with cervical cancer. The inverse correlation of RIP140 with OS of patients is significant in squamous cell carcinoma of the cervix (p=0.015) and not in adenocarcinoma samples but this could be due to the smaller number of cases. A positive correlation between nuclear RIP140 and LCoR expression was demonstrated. Differentiated Kaplan-Meier analysis of RIP140 showed that RIP140 was no longer a negative prognosticator in cervical carcinoma if LCoR nuclear expression was low. Hence, joint expression of both transcription factors RIP140 and LCoR in cervical tissue is associated with a worse prognosis. Of importance, nuclear RIP140 levels (together with histological subtype, tumor size and nodal status) is an additional independent parameter which prognosticated survival in the tested cervical cancer cohort.

One limitation of this study is that the study is a retrospective which analyses the data of the patients who had undergone surgery for cervical cancer from 1993 until 2002. The advantage of a retrospective study is that this enables a long follow-up period, however, therapy options have been modified in the meantime which can further have an influence on the follow-up period. Additionally, the whole patient cohort originates from a single hospital and, for a more detailed analysis, a multi-centre study should be carried out.

In conclusion, RIP140 and LCoR transcription factors may lead to the progression of cervical cancer, and possibly represent novel therapeutic targets for the treatment of this malignancy. Further studies are required to analyze their roles in the biology of cervical cancer and, more precisely, their interaction with p53, E6 and histone proteins. Additionally, the mechanisms of how RIP140 and LCoR interact with other pathways in order to influence the development of cervical cancer have to be studied. Genome-wide profiling of RIP140 and LCoR binding sites in cervical cancer cells will be needed to examine these different cross-talks. In addition, because there is a direct correlation between RIP140 and LCoR with the histone protein modifications H3K4me3 and H3K9ac, analysis of their involvement in the maintenance of the epigenome should be investigated in cervical cancer.

## MATERIALS AND METHODS

### Characteristics of patients and biopsies

Formalin fixed paraffin embedded samples of all assessable cervical cancer cases (250 patients, all without distant metastasis (pM0) at the time point of primary surgery) who had undergone surgery at the Department of Gynecology and Obstetrics, Ludwig-Maximilians-University Munich, Germany, from 1993 until 2002 were included in the study. All patients who had undergone surgery for the treatment of cervical cancer and where the paraffin-embedded tumor was available were included in the study. There was no pre-selection of the patients. Histopathological tumor subtypes were assigned according to the WHO criteria by a gynecological pathologist. Squamous cell carcinoma (SCC) (202 cases) and cervix adenocarcinoma (48 cases) were included in the cohort (Table 4). Other histological subtypes were excluded from the study as there were only few cases. Clinical and follow-up data regarding patient age, OS, tumor size, lymph node status, FIGO classification, tumor grade and tumor subtype were retrieved from the Munich Cancer Registry (Table 4). Median age of patients was 47.0 years (range 20-83 years). Tumor grade included grade I (well differentiated), grade II (moderately differentiated) and grade III (poorly differentiated). In total, five patients received an adjuvant chemotherapy.

**Table 4:** Description of the cohort clinical pathological variables of the patients

	Number (total number of patients: n=250)	%
**Age, years**		
< 49	139	55.6
≥49	111	44.4
**Tumor subtype**		
Squamous	202	80.8
Adenocarcinoma	48	19.2
**Tumor size, pT**		
pT1	110	44.0
pT2	128	51.2
pT3/4	9	3.6
NA	3	1.2
**Tumor grade**		
I	21	8.4
II	143	57.2
III	78	31.2
NA	8	3.2
**FIGO**		
I	64	25.6
II	48	19.2
III	37	14.8
IV	7	2.8
NA	94	37.6
**Number of positive nodes**		
0	151	60.4
≥ 1	97	38.8
NA	2	0.8
**Progression** (over 235 months)		
None	210	84.0
At least one	21	11.6
NA	19	7.6
**Survival** (over 235 months)		
Censured	190	76.0
Dead	49	19.6
NA	11	4.4

### Ethical approval and informed consent

All procedures involving human participants were in accordance with the ethical standards of the institutional and/or national research committee and with the Helsinki declaration of 1964 and its later amendments or comparable ethical standards. The study was approved by the local ethics committee of the Ludwig-Maximilians University of Munich (reference number 259-16, 2016).

### Immunohistochemistry

Expression of RIP140 and LCoR was immuno-histochemically quantified from the embedded cervical cancer samples. Tissue samples were fixed in neutral-buffered formalin (3.7%) straight after resection and then underwent standardized paraffin embedding. For immunohistochemistry, formalin-fixed paraffin-embedded tissue sections (3μm) were first deparaffinised in xylol, rehydrated in a descending ethanol gradient and then prepared for epitope retrieval in a pressure cooker using sodium citrate buffer (pH 6.0). Next, sections were blocked with 3% H_2_O_2_ in methanol at room temperature for 20 min in order to inactivate the endogenous peroxidase. Blocking solution was applied for blocking of the non-specific binding of the primary antibodies. Sections were then consecutively incubated with the following primary antibodies: anti-RIP140(polyclonal rabbit IgG, Sigma Aldrich, St. Louis, USA) and anti-LCoR (polyclonal rabbit IgG, Novus Biologicals, Littleton, USA). Antibody reactivity was analysed using the ZytoChemPlus HRP Polymer System (mouse/rabbit) (Zytomed Systems, Berlin, Germany) according to the manufacturer's protocol. Next, substrate and chromogen (3,3′-diaminobenzidine DAB; Dako, Glostrup, Denmark) were added to the slides, which were then counterstained with Mayer's acidic haematoxylin and cover slipped. Appropriate positive controls (placenta samples) and negative controls (negative control serum added on the placenta: Negative Control for Super Sensitive Rabbit Antibodies, Rabbit IgG, Biogenics, Fremont, USA) were included in each experiment. Nuclear as well as cytoplasmic RIP140 and LCoR staining was then correlated with cytoplasmic staining of E6, nuclear p53 (wild type and mutated on Ser20), H3K9ac and H3K4me3, which has been carried out for former publications [37] [8]. Most of the mutations of p53 destroy the ability of the protein to bind to its target DNA and thereby prevent transcriptional gene activation. In a recent study, we detected a high mutation rate of TP53 in a cervical cancer type where p53 is initially inactivated when cervical cancer develops [37]. The mutation could be correlated with a better OS, presumably due to a better response to therapy.

### Signal quantification

Cervical cancer sections were examined by two independent observers using a Leitz Diaplan microscope (Leitz, Wetzlar, Germany). For each slide, staining was quantified by applying the semiquantitative immunoreactive score (IRS) which is used for optical assessment of the intensity and distribution pattern of antigen expression [43]. The IRS was calculated by multiplying the number of positively stained cells (in %) (0: no staining; 1: ≤10% of the cells; 2: 11% to 50%; 3: 51% to 80%, 4: >80%) with staining intensity (0: none; 1: weak; 2: moderate; 3: strong). We used a scale from 0-1 (no expression) to 12 (very high expression). A receiver operating characteristic curve (ROC-curve) was used to determine the cut-off level between RIP140 and LCoR overexpression and reduced RIP140 respectively LCoR expression. For identification of the cut-off level for RIP140 and LCoR, the maximum difference between sensitivity and specificity was used. Images were taken with a CCD color camera (JVC, Victor Company of Japan, Japan).

### Statistics

IBM SPSS Statistics for Windows, Version 22.0. Armonk, NY: IBM Corp. was used for data analysis. P-values lower than p<0.05 were considered statistically significant. Survival times were compared by Kaplan-Meier analysis, and differences in the OS times of patients were tested for significance by the Cox-Mantel log-rank test. Group comparisons of independent groups regarding ordinal analysis variables were tested with the Mann-Whitney U test or the Kruskal-Wallis test as appropriate. All histopathological variables which had been documented, have been tested in the multivariable analysis. Correlations between ordinal variables were tested using Spearman's rank correlation coefficient. Cox regression analysis was used to compare the risk of death in patients with and without RIP140 and LCoR expression when the effects of further factors were accounted for. Independent variables included in the Cox regression model were RIP140 and LCoR expression, age, tumor size (pT) (T1-T4), histological subtype (Squamous cell carcinoma and Adenocarcinoma), tumor grade (G1, G2, G3), FIGO classification (Stage I-IVB) and lymph node status (pN) (pN0=no regional lymph node metastasis, pN1=regional lymph node metastasis). We used neither forward nor backward variable selection because all stepwise procedures have strongly be criticized [44, 45]. Variables were therefore selected based on theoretical considerations and forced into the model.

## SUPPLEMENTARY FIGURE



## Abbreviations

ERα: Estrogen receptor α
H3K4me3: Histone H3 trimethyl K4
H3K9ac: Histone H3 acetyl K9 H3K9ac
LCoR: Ligand dependent corepressor
NRIP1: Nuclear Receptor Interacting Protein 1
pN: Nodal status
pT: Tumor size
OS: Overall survival
RIP140: Receptor Interacting Protein of 140 kDa
SCC: Squamous cell carcinoma
