# Supplementary material for: Investigation of RIP140 and LCoR as independent markers for poor prognosis in cervical cancer
Source: Oncotarget. 2017 Oct 31;8(62):105356–71. doi: 10.18632/oncotarget.22187 (PMC5739643; doi:10.18632/oncotarget.22187)
Supplement: Supplementary file 1 [file oncotarget-08-105356-s001.pdf]

Investigation of RIP140 and LCoR as independent markers for poor prognosis in cervical cancer

SUPPLEMENTARY MATERIALS

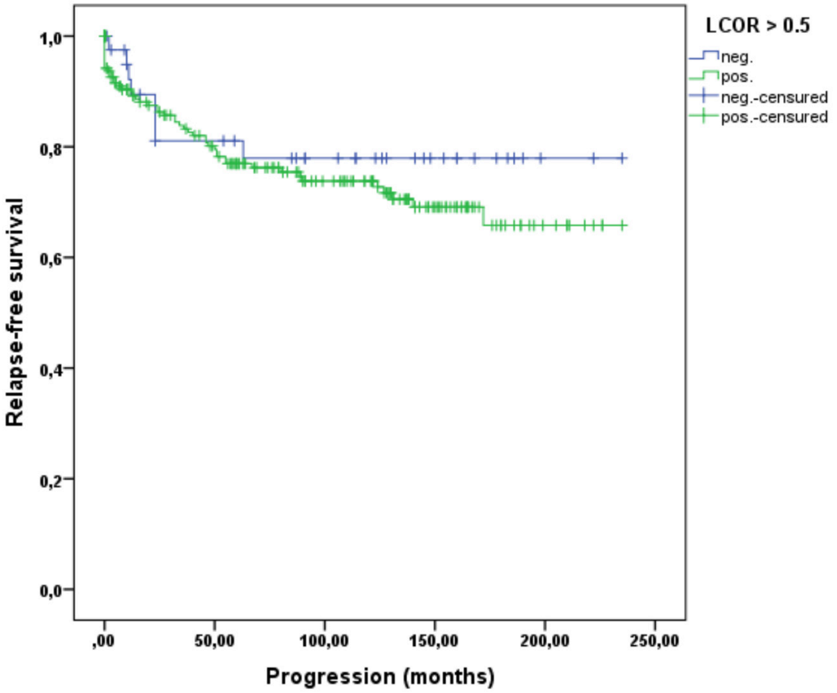

Supplementary Figure 1: Relapse-free survival in patients with nuclear LCoR IRS>0.5 expression. A trend for a shorter relapse-free survival can be seen in patients with nuclear LCoR IRS>0.5 expression in the primary cervical tumor (p=0.081).
